# Parents’ satisfaction with physiotherapy services for neuropediatric outpatients in government and private hospitals in the United Arab Emirates: a cross-sectional study

**DOI:** 10.12688/f1000research.151041.1

**Published:** 2024-07-04

**Authors:** Hanadi Ahmed AlNaqbi, Meeyoung Kim, Nabeel Al-Yateem, Fatma Hegazy

**Affiliations:** 1 Department of Physiotherapy, College of Health Sciences, University of Sharjah, Sharjah, Sharjah, United Arab Emirates; 2 Laboratory of Health Science & Nanophysiotherapy, Department of Physical Therapy, Graduated School, Yongin University, Yongin, South Korea; 3 Neuro musculoskeletal Rehabilitation Research Group, Research Institute of Medical and Health Science, University of Sharjah, Sharjah, Sharjah, United Arab Emirates; 4 Department of Nursing, College of Health Sciences, University of Sharjah, Sharjah, Sharjah, United Arab Emirates; 5 Department of Paediatrics Physical Therapy, Faculty of Physical Therapy, Cairo University, Giza, Giza Governorate, Egypt

**Keywords:** Parents satisfaction, Physiotherapy, Pediatric rehabilitation, Neurological disabilities

## Abstract

**Background:**

Healthcare, like other industries, emphasizes performance, quality, and consumer experience while also attempting to reduce costs. However, high-quality healthcare remains paramount for vulnerable and ill patients. This study aimed to investigate parents' and caregivers' level of satisfaction with physiotherapy services provided to neuropediatric outpatients on the United Arab Emirates (UAE).

**Methods:**

This descriptive cross-sectional study included 103 parents/caregivers of children with neurological disabilities that were randomly selected from different Emirates Health Services Hospitals in the UAE. Data was collected using the long-form Patient Satisfaction Questionnaire (PSQ-III).

**Results:**

The overall mean satisfaction was 159±7.73 (out of 250 points). Communication (20.36/25), interpersonal factors (20.17/35), and doctor-patient time (20.17/35) had the highest mean satisfaction scores (8.06/10). The lowest mean satisfaction scores were for access/availability/convenience (34.60/60), technical quality (33.17/50), and economic elements (23.83/40).

**Conclusion:**

Despite participants’ overall satisfaction scores being positive, some service domains require improvement to improve satisfaction, specifically the access/availability/convenience, technical quality, and economic elements. These areas should be prioritized by service providers and managers to improve patients’ experiences and clinical outcomes.

## Introduction

Many industries worldwide, including healthcare, have increasingly prioritized performance, quality, and user satisfaction while simultaneously attempting to reduce costs. Healthcare providers deliver vital services to vulnerable and critically ill patients, along with essential preventative and health promotion services. Several healthcare organizations worldwide have launched quality improvement initiatives to improve patient satisfaction and prevent negative experiences, but it is becoming increasingly important to implement effective strategies to provide quality care that is also cost-efficient. ^1^^,^^2^

Satisfaction is known as a belief and attitude regarding certain or specific services provision of an institution. ^3^^,^^4^ Patient satisfaction is considered the most important indicator for health service quality assessment. ^5^ Parental and patient satisfaction become a well-established outcome indicator for assessing the quality of healthcare system. ^4^^,^^6^ Parental satisfaction is determined by how effectively parents ‘expectations of ideal care are balanced with how they actually feel about real available care. ^7^^,^^8^ According to the numerous studies, poor communication and lack of information are the most serious parent/patient complaint. ^9^^,^^10^ Even when therapists try to explain the patient condition, their terminology is often misunderstood. So, Listening to parents is very important in process of communication. ^11^

Among the most widely used patient satisfaction tool is the Hospital Consumer Assessment of Healthcare Providers and Systems (HCAHPS) survey. Many organizations use the HCAHPS as a key performance indicator. ^12^ However this tool is general and nature and is of limited use for specific and specialized services like the physiotherapy service. ^13^ Consistent with global trends, the United Arab Emirates (UAE) has prioritized the development of a world-class healthcare system to serve its population. To achieve this, the UAE government introduced new quality criteria and mandated that all healthcare institutions receive accreditation from national and international agencies. ^14^

Furthermore, healthcare organizations in the UAE consistently undergo evaluation against comprehensive quality outcomes to assess their efficiency and determine funding and reimbursement. For example, the Department of Health in Abu Dhabi uses a healthcare quality index called “Muashir” to measure the performance of healthcare organizations against pre-set key performance indicators. Distinguished facilities receive a rating from one to five domains, which is made public and used to allocate funding to these institutions. ^15^ This system means that the operation of these healthcare organizations depends on their achievement of quality outcomes, which results in competition among organizations to perform better and attract more funding. This approach is not implemented universally across the UAE, as healthcare services are managed federally through the Ministry of Health and Prevention (MOHAP) or locally by local health authorities. However, many local health authorities have recognized the successful implementation of Muashir and are considering rolling out this approach across their healthcare institutions.

To support physiotherapists to make a maximum contribution to developing healthcare services for their patient population in the UAE, elements of their practice that contribute to patient satisfaction need to be identified. Identifying these elements can form the basis for planning and implementing relevant measures to improve available services. At the clinical level, physiotherapy patients who are satisfied with their treatment tend to interact well with their therapist, remain loyal to them, and seek further treatment when necessary. ^16^

When initiating treatment, physiotherapists meet with their patients (and patients’ parents/caregivers in pediatric cases) for a consultation to develop an appropriate treatment plan. ^17^ The fulfilment of this plan should lead to patient satisfaction; however, many systemic, process, and outcome factors could affect the execution of the treatment plan and thereby affect patient satisfaction. This means it is essential to clarify aspects that impact patient satisfaction in these services. Therefore, the present study explored patient satisfaction in the context of rehabilitation healthcare (physiotherapy) services in the UAE, as the principal investigator is a clinician working in pediatric rehabilitation services. This study was conducted to determine parents’/caregivers’ level of satisfaction with the physiotherapy services provided to their children in the physiotherapy departments of private and government hospitals in the UAE.

## Methods

### Study design

This study used a cross-sectional correlational design. Participants completed self-report questionnaires that assessed the satisfaction of parents/caregivers of children attending rehabilitation services and other relevant variables.

### Participants

We included Parents or caregivers of children that had physical disabilities and neurological problems who were receiving rehabilitation sessions in physiotherapy departments in government and private hospitals in the UAE. Parents/caregivers were invited to participate if they were able to read and understand English and had a child aged between 5 months and 16 years that was a neuropediatric patient who had received at least 12 physiotherapy sessions. Parents and caregivers of patients who were admitted to hospital (inpatients) were excluded from this study. In addition, we did not include patients from all physiotherapy specialties as their needs may differ, which would impact their satisfaction.

Convenience sampling was used to recruit parents/caregivers of children receiving rehabilitation services. All major hospitals for children’s rehabilitation governed by the Emirates Health Services authority were contacted to obtain approval to conduct this study. Those settings that responded and approved this study were approached to assist with recruitment and all eligible parents/caregivers in these settings were invited to participate. Data were collected from 103 parents/caregivers of children with various neurological disabilities attending rehabilitation services at different Emirates Health Services facilities.

The sample size was calculated depending on the proportion (78%) stated in a previous study addressing the same research question [48]. The appropriate sample size with sufficient power of 80% (alpha= 0.05) was calculated by the Epi Info, a program developed by the Centers for Disease Control and Prevention.

### Data collection procedure

The long form of the Patient Satisfaction Questionnaire (PSQ-III) was used in this study (English language version), which was validated in previous studies. ^18^^,^^19^ The scale is open access and available for researchers use through the authors website. ^20^ The scale was found to be reliable, with Cronbach’s alpha coefficients of 0.92 for the whole scale and 0.74–0.89 for the subscales. ^17^ The PSQ-III is a 50-item survey that measures global satisfaction with medical care as well as satisfaction with seven specific aspects of care: general satisfaction, technical quality, interpersonal manner, communication, financial aspect of care, time spent with doctor, and accessibility of care.

Respondents are asked to rate their satisfaction with the medical care they received, with responses on a scale from 1 to 5 (strongly agree to strongly disagree) that indicate to what extent they agreed with each statement. ^18^ An open-ended comments option was added at the end of the structured questionnaire to accommodate qualitative information from participants regarding their suggestions for service improvements. We also collected information on the children’s age, gender, and the duration they had received physiotherapy treatment.

Participants were parents or caregivers who usually accompanied children to their physiotherapy session. They were selected following contact with the heads of the physiotherapy departments in different government and private hospitals via electronic communications that explained the study purpose. A link to the electronic questionnaire (PSQ-III) was distributed to eligible parents/caregivers through their preferred communication channel.

### Ethical consideration

Ethical approval was obtained from the Research Ethics Committee of the University of Sharjah [approval no: REC-21-04-28-02-S], and the ethical committee of the Ministry of Health and Prevention [Approval Reference No: MOHAP/DXB-REC/JJJ/No. 57/2021] on April 2021. Before recruiting participants, written informed consent which included information of the study’s purpose, was obtained from all eligible participants. Each parent who expressed interest to participate in this study was instructed to read, understand, and sign the consent form. Furthermore, the integrity and confidentiality of all data collected during the study were assured by local institutional policies. Participants were informed that they had their right to withdraw at any time without prejudice and no incentives were offered at any point in the study.

### Statistical analysis

Data were analyzed using SPSS Software. Descriptive statistics were used to describe participants’ demographics and levels of satisfaction. Continuous variables were expressed as means and standard deviation, while categorical variables were expressed as counts and percentages. Correlational analysis was performed to assess the association between demographic variables and the satisfaction score.

## Results

Before proceeding with data analysis, the reliability of the study questionnaire was assessed to ensure valid and reliable results. The reliability of the scale used in this study was assessed and returned a Cronbach’s alpha value of 0.7, which is considered acceptable reliability value.

### Sociodemographic characteristics

In total, 103 parents/caregivers participated in this study, of which 44.7% (n=46) had female children receiving rehabilitation and the remaining (55.3%, n=57) had male children receiving rehabilitation. The majority of participants had children that were scheduled for a long duration of physiotherapy treatment (i.e., >12 sessions). The mean age of the children was 57±48 months (4.75 years). Table 1 presents sociodemographic details of participants’ children.

**Table 1.** Sociodemographic characteristics of participants' children.

		Count	%
Child’s sex	Girl	46	44.7
	Boy	57	55.3
Duration of the child’s physiotherapy treatment	<12 sessions	3	2.9
	>12 sessions	100	97.1
Child’s age (months)	Mean	57	
	Standard deviation	48	

### Participants' satisfaction with seven aspects of care

The mean satisfaction score for the sample was 159±7.73 (out of 250 points). The highest levels of satisfaction were found for communication (20.36/25), followed by interpersonal aspects (20.17/35) and time spent with the doctor (8.06/10). The lowest mean satisfaction scores were recorded for access/availability/convenience (34.60/60, SD=3.08), technical quality (33.17±1.97/50) and financial aspects (23.83±2.66/40). Table 2 presents the descriptive statistics for the survey items.

**Table 2.** Total and domain satisfaction scores.

	Mean	Standard deviation	Minimum	Maximum
Total satisfaction (max. score = 250)	159.49	7.73	142.00	186.00
General satisfaction (max. score = 30)	19.28	1.92	14.00	24.00
Technical quality (max. score = 50)	33.17	1.97	26.00	38.00
Interpersonal aspects (max. score = 35)	20.17	1.18	17.00	24.00
Communication (max. score = 25)	20.36	1.07	18.00	24.00
Financial aspects (max. score = 40)	23.83	2.66	18.00	30.00
Time spent with doctor (max. score = 10)	8.06	0.68	6.00	10.00
Access/availability/convenience (max. score = 60)	34.60	3.08	26.00	41.00

Table 3 shows the level of satisfaction with each PSQ-III item. In the general satisfaction domain, most (97.1%) participants agreed that some aspects of the medical care that they received could be improved. However, most (94.2%) participants were satisfied that their child was treated by medical staff who were aware of the latest medical developments. Regarding the interpersonal aspect domain, most (90%) participants indicated that the doctors always tried to prevent them worrying. In terms of communication, most (89.3%) participants reported that when they visited the doctor, they were always free to express concerns and believed that this was important. A majority (68.9%) of participants sometimes worried about having to pay a large medical bill. In addition, most (88.3%) participants reported that the doctors usually spent plenty of time with their children, and 88.3% noted that they could easily get hospital care without any trouble.

**Table 3.** Satisfaction scores for all items.

Item	Agree n (%)	Uncertain n (%)	Disagree n (%)
I am very satisfied with the medical care my child received	97 (94.2)	4 (3.9)	2 (1.9)
There are some things about the medical care my child received that could be better	100 (97.1)	2 (1.9)	1 (1.0)
All things considered, the medical care that my child received is excellent	71 (69)	21 (20.4)	11 (10.6)
There are things about the medical care my child received that need to be improved	94 (91.3)	4 (3.9)	5 (4.9)
The medical care my child received is just about perfect	85 (82.5)	7 (6.8)	11 (10.7)
I am dissatisfied with some things about the medical care my child received	20 (19.4)	0 (0.0)	83 (80.6)
When my child goes for medical care, they are careful to check everything when treating and examining them	90 (87.3)	3 (2.9)	10 (9.7)
The therapist needed to be more thorough in treating and examining my child	103 (100)	0 (0.0)	0 (0.0)
I think my doctor’s office has everything needed to provide complete medical care	78 (75.7)	4 (3.9)	21 (20.4)
The physiotherapist is good about explaining the reason for physiotherapy treatment	57 (55.3)	18 (17.5)	28 (27.2)
The medical staff that treated my child know about the latest medical developments	102 (99.1)	1 (1.0)	0 (0.0)
Some of the doctors my child has seen lacked experience with my child’s condition	3 (2.9)	6 (5.8)	94 (91.3)
My child’s physiotherapist is very competent and well-trained	94 (91.3)	9 (8.7)	0 (0.0)
I have some doubts about the ability of the doctors who treated my child	4 (3.9)	3 (2.9)	96 (93.2)
The physiotherapist answered all my questions	103 (100.0)	0 (0.0)	0 (0.0)
The therapist rarely gave me advice about ways my child could avoid further problems and stay healthy	5 (4.9)	1 (1.0)	97 (94.1)
The physiotherapist acted business-like and impersonal toward my child	1 (1.0)	0 (0.0)	102 (99.1)
The therapist always does their best to keep me from worrying	103 (100.0)	0 (0.0)	0 (0.0)
When my child receives medical care, the physiotherapist should pay more attention to our privacy.	101 (98.1)	0 (0.0)	2 (2.0)
During my child’s initial visit, the physiotherapist took time to listen to my explanation about my child’s condition	103 (100.0)	0 (0.0)	0 (0.0)
The doctors who treated my child have a genuine interest in my child as a person	2 (1.9)	0 (0.0)	101 (98.1)
The physiotherapist treated my child in a very friendly and courteous manner	103 (100.0)	0 (0.0)	0 (0.0)
The physiotherapist who treated my child should give us more respect	103 (100.0)	0 (0.0)	0 (0.0)
The physiotherapist was good about my child’s condition	103 (100.0)	0 (0.0)	0 (0.0)
Sometimes doctors used medical terms without explaining what they meant	2 (1.9)	0 (0.0)	101 (98.1)
During my child’s medical visits, I am always allowed to say everything that I think is important	100 (97.1)	3 (2.9)	0 (0.0)
The physiotherapist sometimes ignored what I told them	1 (1.0)	0 (0.0)	102 (99.1)
The physiotherapist listened carefully to what I had to say	102 (99.0)	1 (1.0)	0 (0.0)
I was allowed to contribute to my child’s care	26 (25.2)	1 (1.0)	76 (73.8)
I feel confident that my child can get the medical care that they need without being set back financially	72 (69.9)	0 (0.0)	31 (30.1)
I worry sometimes about having to pay large medical bills	26 (25.2)	0 (0.0)	77 (74.8)
Regardless of the health problems my child has now or develops later, I feel protected from financial hardship	46 (44.7)	0 (0.0)	57 (55.3)
Sometimes it is a problem to cover my share of costs for a medical care visit	48 (46.6)	0 (0.0)	55 (53.4)
I feel insured and protected financially against all possible medical problems	42 (40.7)	1 (1.0)	60 (58.3)
I have to pay for more medical care that I can afford	66 (64.1)	0 (0.0)	37 (35.9)
The amount I have to pay to cover my child’s medical care needs is reasonable	20 (19.4)	2 (1.9)	81 (78.6)
The physiotherapist usually spends plenty of time with my child	101 (98)	2 (1.9)	0 (0.0)
Those who provide medical care sometimes hurried too much when they treated my child	5 (4.9)	0 (0.0)	98 (95.1)
If my child needs a physiotherapy appointment, I can get one without any trouble	91 (88.3)	4 (3.9)	8 (7.8)
The physiotherapist gave my child appointments for periods that would reduce the waiting time	86 (83.5)	0 (0.0)	17 (16.5)
It is easy for my child to get physiotherapy care	89 (86.4)	0 (0.0)	14 (13.6)
The office where my child gets medical care should be open for more hours than it currently is	87 (84.5)	1 (1.0)	15 (14.6)
The place where my child gets medical care is very conveniently located	66 (64.2)	21 (20.4)	16 (15.6)
When my child gets a medical care, we have to wait too long	10 (9.7)	2 (1.9)	91 (88.4)
If I have a medical question, I can reach a physiotherapist without any problem	99 (96.1)	4 (3.9)	0 (0.0)
I find it hard to get an appointment for my child’s medical care right away	65 (63.2)	0 (0.0)	38 (36.9)
The office hours when my child can get medical care are convenient for me	61 (59.3)	0 (0.0)	42 (40.8)
I am usually kept waiting for long time when I am in the physiotherapist’s office	6 (5.8)	4 (3.9)	93 (90.3)
The physiotherapist was available for discussion with me on my child’s condition on regular basis	70 (68.0)	0 (0.0)	33 (32.0)
I am able to get medical care whenever my child needs it	76 (73.8)	1 (1.0)	26 (25.2)

We also assessed the correlations between the child's age and parents' /caregivers' satisfaction scores. These variables showed a significant, negative, and weak correlation, which indicated that satisfaction tended to decrease as the child’s age increased. A nonparametric Mann-Whitney U test was used to assess differences in parents’/caregivers’ satisfaction scores by the child’s sex and physiotherapy duration. This analysis showed that there were no significant differences between these groups in terms of their satisfaction scores.

## Discussion

Parents' and caregivers' satisfaction with their child’s rehabilitation intervention is necessary for the sustainability of services. Conducting patient satisfaction surveys can help enhance clinical practice, and can also provide relevant data for the marketability of healthcare services and quality of care delivered. ^19^^,^^21^ Patients are considered users of healthcare services, meaning patient satisfaction is an important tool to evaluate the quality of services provided. In this study, the overall level of satisfaction with neuropediatric rehabilitation services was high, but a lower level of satisfaction was observed when specific aspects of care were assessed. This suggested that it is not possible to assess the level of patient satisfaction by simply asking patients if they were happy with the services that they received.

We found no significant differences in parents’/caregivers’ satisfaction by the child’s sex and physiotherapy duration. However, this finding may be because our sample size was insufficient to allow these comparisons. Similarly, a previous study revealed patient satisfaction was not affected by their age, educational status or occupation. ^22^ In contrast, other studies showed that age, sex, ethnicity, education, and occupation were the main predicators of overall patient satisfaction. For example, a study from Nigeria reported that the communication and access/availability/convenience domains were strongly associated with sex; however, there was no sex-based difference in satisfaction with the time spent with doctor, technical quality, interpersonal financial, and general satisfaction domains. ^23^

However, we found that age was the best predictor of satisfaction in the majority of satisfaction dimensions, whereas the Nigerian study reported patients’ age was not a determinant of their satisfaction with services they received. ^23^

In the present study, the satisfaction of parents\caregivers differed across various areas examined in the questionnaire. The mean total satisfaction score (63.8%, 159.49/250) showed that the majority of participants had a good level of satisfaction with the physiotherapy treatment that their children received at the government and private hospitals run by Emirates Health Services. A previous study reported the mean overall score for patient satisfaction with physiotherapy care was 68.2%, which was consistent with our results. ^24^ However, other studies reported lower levels of satisfaction for various reasons. A study from Nepal found that patients’ general satisfaction was relatively low (39%), although this was attributed to the unavailability of certain desired facilities. ^25^ Participants in our study were satisfied with the time the therapist spent with their child (80.6%) in each session. This finding was expected because therapists in the participating services typically spend 45–60 min in one treatment session with their patients. Our participants indicated that their satisfaction was increased when the therapist spent a long time with their child and explained the treatment plan.

In contrast to our results, a study from Dubai reported that 40% of parents/caregivers were dissatisfied with the time that their children spent with the therapist. Those authors suggested that this dissatisfaction could be explained by the therapists’ heavy workloads or the patients’ condition not requiring a long time for treatment. ^26^

Another study conducted in Katsina State, Nigeria, found patients reported low satisfaction with the time spent with the doctor because there was a large number of surgeries completed and patients felt they did not receive proper interaction. ^23^

Previous studies focused on psychological issues related to pediatric practice revealed that parents/caregivers were interested in knowing more about their children’s psychological problems and good communication was a powerful predictor of satisfaction. Good communication during medical visits was reported to significantly influence parents’/caregivers’ satisfaction, functional status, and patient compliance. ^27^ This was consistent with our finding that most (81.4%) participants were satisfied with the therapist because there was good communication between the therapist and parents/caregivers. This was consistent with the results of other studies that found the highest level of satisfaction was reported for the communication domain. ^28^ One study suggested that communication had the highest mean satisfaction score because patients appreciated their therapists’ listening ability. ^24^

The interpersonal and access/availability/convenience domains had the lowest scores in our study (57.6% and 57.7%, respectively). This was consistent with a previous study that also showed these two domains had the lowest mean satisfaction scores. ^23^ Financial aspects also had a low satisfaction score in our study (59.6%) Some of our participants were not satisfied with the financial domain because they felt that they were not protected from financial hardship. Others noted they were required to pay for services from their own pocket, which could expose them to future financial issues.

We faced some challenges that might have affected this study, particularly parents’/caregivers’ reluctance to complete the questionnaire and the small sample size because of the COVID-19 pandemic (Coronavirus disease of 2019). In addition, the questionnaire was not translated into Arabic, which makes it difficult to generalize the findings and might have contributed to the small sample size. However, a reasonable number of parents/caregivers completed the survey, and our findings may help in identifying reasons for dissatisfaction with neuropediatric rehabilitation services, as well as which domains require improvement and what improvement they may need.

## Conclusions

In conclusion, this study sheds light on the crucial aspect of parents' satisfaction with physiotherapy treatment for neuropediatric outpatients in the UAE. The findings underscore the overall positive satisfaction reported by parents and caregivers regarding various aspects of physiotherapy services, particularly in communication, interpersonal factors, and doctor-patient time. However, it is evident that there are areas in need of improvement, notably access/availability/convenience, technical quality, and economic elements. These findings emphasize the importance of continuous assessment and enhancement of healthcare services to meet the evolving needs of patients and their families. Addressing the identified areas of concern is paramount to enhancing patient experiences and ultimately improving clinical outcomes. Therefore, it is imperative for service providers and managers to prioritize these domains in their efforts to optimize the quality of care provided to neuropediatric outpatients and ensure the delivery of patient-centered healthcare in the UAE.

## Data Availability

Harvard Dataverse: “Replication Data for: Parents’ satisfaction with physiotherapy services for neuropediatric outpatients in Government and Private Hospitals in the United Arab Emirates: a cross-sectional study” https://doi.org/10.7910/DVN/QBANIG. ^29^ The data set contains the underlying data:
• Satisfaction data - with items recoded.tab: This file contains the socio-demographic and QoL variables. Data are available under the terms of the Creative Commons Zero “No rights reserved” data waiver (CC0 1.0 Public domain dedication).
