# Supplementary material for: Parents’ satisfaction with physiotherapy services for neuropediatric outpatients in government and private hospitals in the United Arab Emirates: a cross-sectional study
Source: F1000Res. 2024 Jul 4;13:739. [Version 1] doi: 10.12688/f1000research.151041.1 (PMC11289589; doi:10.12688/f1000research.151041.1)
Supplement: Peer review history

# Reviewer report: Reviewer response for version 1

**DOI:** 10.5256/f1000research.165661.r300260

**Published:** 2024-07-30

**Authors:** Ehab Mohamed Abd El-Kaf

**Affiliations:** 1 Umm Al Qura University, Mecca, Saudi Arabia

This manuscript makes a valuable contribution to understanding parental satisfaction with physiotherapy services for children with neurological disabilities in the UAE. The study highlights the local importance and relevance of this issue and provides useful insights for healthcare providers seeking to improve service quality. Overall, this manuscript provides a comprehensive overview of parental satisfaction with physiotherapy services for children with neurological disabilities in the UAE. Enhancing the introduction with additional references, clarifying secondary objectives, and providing more details on the sampling process and subgroup analyses would further improve the manuscript.

 Here are a detailed review of the sections.

 1. The introduction is clear and effectively sets the stage for the study, emphasizing the importance of patient satisfaction in healthcare within the UAE's evolving landscape. While the background information on patient satisfaction is comprehensive, adding recent studies on similar settings would enhance this section.

 2. The goals and objectives of the study are well-stated and align with the introduction. The aim to investigate parents' satisfaction with physiotherapy services for neuropediatric patients is clear. However, clarifying any secondary objectives would provide a more complete picture of the study's scope.

 3. The methods section is detailed and well-organized, outlining the study design, participant recruitment, data collection, and analysis procedures. The use of the Patient Satisfaction Questionnaire (PSQ-III) is well-justified, and ethical considerations are thoroughly addressed. More details on the sampling process, including selection criteria and potential biases, would improve transparency and replicability.

 4. Results are presented clearly with tables that effectively illustrate key findings. The mean satisfaction scores for different service domains are well-documented, and the statistical analysis is sound. Including more detailed demographic data and subgroup analyses would provide additional context and highlight factors influencing parental satisfaction.

 5. The discussion interprets the results well, relating them to existing literature and emphasizing the study's local significance. Identifying areas for improvement, such as access, technical quality, and economic elements, is valuable. The discussion could be enriched by exploring strategies for addressing these areas and discussing the implications for policy and practice in more detail.

 6. Comparing the findings with similar studies in other regions would offer a broader perspective.

 7. The conclusion succinctly summarizes the main findings and their implications, emphasizing the need for ongoing assessment and improvement of physiotherapy services. Including specific recommendations for future research and practice would strengthen the conclusion.

Is the work clearly and accurately presented and does it cite the current literature?

Yes

If applicable, is the statistical analysis and its interpretation appropriate?

Yes

Are all the source data underlying the results available to ensure full reproducibility?

Yes

Is the study design appropriate and is the work technically sound?

Yes

Are the conclusions drawn adequately supported by the results?

Yes

Are sufficient details of methods and analysis provided to allow replication by others?

Partly

Reviewer Expertise:

Professor of Physical Therapy.

I confirm that I have read this submission and believe that I have an appropriate level of expertise to confirm that it is of an acceptable scientific standard.

# Reviewer report: Reviewer response for version 1

**DOI:** 10.5256/f1000research.165661.r300265

**Published:** 2024-07-12

**Authors:** Bhamini Krishna Rao

**Affiliations:** 1 Physiotherapy, Manipal College of Health Professions, Manipal Academy of Higher Education, Manipal, Karnataka, India

The manuscript titled "Parents’ satisfaction with physiotherapy services for neuropediatric outpatients in government and private hospitals in the United Arab Emirates: a cross-sectional study" presents a valuable exploration of parental satisfaction with physiotherapy services for children with neurological disabilities in the UAE. The study design, which utilizes a cross-sectional correlational approach, is appropriate for the research objectives and provides a comprehensive overview of the satisfaction levels among parents and caregivers.

 The methods section is detailed and well-structured, clearly outlining the study design, participant recruitment, data collection, and analysis procedures. The choice of the Patient Satisfaction Questionnaire (PSQ-III) is justified and its reliability is well-documented, making it a suitable tool for this study. The ethical considerations are thoroughly addressed, ensuring the integrity and ethical soundness of the study. However, providing more details on the sampling process, including the selection criteria and any potential biases, would enhance the transparency and replicability of the methodology.

 The results are presented clearly and concisely, with comprehensive tables that effectively illustrate the key findings. The analysis is robust, and the interpretation of the data is logical and consistent with the study's objectives. The sociodemographic characteristics of the participants are well-documented, providing important context for understanding the results. The correlation analysis between demographic variables and satisfaction scores is particularly useful, highlighting the factors that influence parental satisfaction. Including more detailed subgroup analyses could provide additional insights into these factors.

 The discussion effectively interprets the results in the context of existing literature, highlighting both the strengths and areas needing improvement in the physiotherapy services. The identification of areas requiring improvement, such as access, technical quality, and economic elements, is particularly valuable for informing future service enhancements. The discussion could be further enriched by exploring potential strategies for addressing these areas and by discussing the implications of the findings for policy and practice in more detail. Additionally, a comparison with similar studies in other regions could provide a broader perspective on the findings and underscore the study's relevance in a global context.

 In conclusion, this study sheds light on the crucial aspect of parents' satisfaction with physiotherapy treatment for neuropediatric outpatients in the UAE. The findings underscore the overall positive satisfaction reported by parents and caregivers regarding various aspects of physiotherapy services, particularly in communication, interpersonal factors, and doctor-patient time. However, it is evident that there are areas in need of improvement, notably access, technical quality, and economic elements. These findings emphasize the importance of continuous assessment and enhancement of healthcare services to meet the evolving needs of patients and their families. Addressing the identified areas of concern is paramount to enhancing patient experiences and ultimately improving clinical outcomes. Therefore, it is imperative for service providers and managers to prioritize these domains in their efforts to optimize the quality of care provided to neuropediatric outpatients and ensure the delivery of patient-centered healthcare in the UAE.

 Suggestions for Improvement:

 The abstract can be reorganized to suit the title of the study by giving importance to parents whose children receive long term rehabilitation services.

 The introduction can emphasize more on how caregiving is difficult in neuropediatric population rather than giving too much importance to general aspects of patient satisfaction

 Provide more details on the sampling process and potential biases in the methods section.

 Include more detailed subgroup analyses in the results section to provide additional insights into factors influencing satisfaction.

 The results section can highlight parents' or caregivers' characteristics and then compare it with the patient satisfaction scores.

 Explore potential strategies for improving areas of low satisfaction in the discussion.

 Compare findings with similar studies in other regions to provide a broader context.

 Include specific recommendations for future research and practice in the conclusion.

 Recommendation: Approve for indexing with minor revisions.

Is the work clearly and accurately presented and does it cite the current literature?

Yes

If applicable, is the statistical analysis and its interpretation appropriate?

Yes

Are all the source data underlying the results available to ensure full reproducibility?

Yes

Is the study design appropriate and is the work technically sound?

Yes

Are the conclusions drawn adequately supported by the results?

Yes

Are sufficient details of methods and analysis provided to allow replication by others?

Yes

Reviewer Expertise:

Pediatric neurorehabilitation, Neonatal intensive care unit , pelvic floor dysfunction and physiotherapy

I confirm that I have read this submission and believe that I have an appropriate level of expertise to confirm that it is of an acceptable scientific standard.
